# Cytomegalovirus, Epstein–Barr virus and risk of breast cancer before age 40 years: a case–control study

**DOI:** 10.1038/sj.bjc.6601822

**Published:** 2004-04-20

**Authors:** A K Richardson, B Cox, M R E McCredie, G S Dite, J-H Chang, D M Gertig, M C Southey, G G Giles, J L Hopper

**Affiliations:** 1 Department of Public Health and General Practice, Christchurch School of Medicine and Health Sciences, University of Otago, PO Box 4345, Christchurch, New Zealand; 2 Department of Preventive and Social Medicine, Dunedin School of Medicine, University of Otago, PO Box 913, Dunedin, New Zealand; 3 Centre for Genetic Epidemiology, University of Melbourne, Level 2, 723 Swanston Street, Carlton, Victoria 3053, Australia; 4 Department of Pathology, University of Melbourne, Victoria 3010, Australia; 5 Cancer Epidemiology Centre, The Cancer Council Victoria, Melbourne, Victoria 3053, Australia

**Keywords:** breast cancer, cytomegalovirus, Epstein–Barr virus, aetiology, epidemiology

## Abstract

We investigated whether there is an association between cytomegalovirus (CMV) and Epstein–Barr virus (EBV) IgG levels and risk of breast cancer before age 40 years. CMV and EBV IgG levels were measured in stored plasma from 208 women with breast cancer and 169 controls who participated in the Australian Breast Cancer Family Study (ABCFS), a population-based case–control study. CMV and EBV IgG values were measured in units of optical density (OD). Cases and controls did not differ in seropositivity for CMV (59 and 57% respectively; *P*=0.8) or EBV (97 and 96% respectively; *P*=0.7). In seropositive women, mean IgG values were higher in cases than controls for CMV (1.20 *vs* 0.98 OD, *P*=0.005) but not for EBV (2.65 *vs* 2.57 OD, *P*=0.5). The adjusted odds ratios per OD unit were 1.46 (95% CI 1.06–2.03) for CMV IgG and 1.11 (0.93–1.33) for EBV IgG. The higher mean CMV IgG levels found in women with breast cancer could be the result of a more recent infection with CMV, and may mean that late exposure to CMV is a risk factor for breast cancer.

It has been hypothesised that some breast cancers might be caused by late exposure (in adulthood rather than in childhood) to a common virus (Richardson, 1997). This is consistent with the geographical distribution of breast cancer. In countries with low incidence, exposure to common viruses such as cytomegalovirus (CMV) and Epstein–Barr virus (EBV) occurs early in childhood and seropositivity is almost invariable before adulthood. In countries with high incidence, seroconversion typically occurs later in life and only 60–70% of adults are seropositive.

In contrast to childhood exposure, which is usually asymptomatic, later exposure to CMV or EBV can cause infectious mononucleosis, a recognisable illness. A case–control study found an increased risk of breast cancer with increasing age at onset of self-reported infectious mononucleosis, and it was suggested that this might be related to delayed exposure to EBV (Yasui *et al*, 2001). Infectious mononucleosis is only a surrogate for delayed CMV or EBV exposure and recall bias may have affected the results. Therefore, to test the hypothesis above it is important to determine the CMV and EBV antibody status of women with and without breast cancer. IgG titres rise initially after infection and then gradually decline, with residual antibody detectable for several years (IARC, 1997; Mendez *et al*, 1999), so IgG levels are higher in people who have had more recent infections.

We tested stored plasma samples from a population-based case–control study of early-onset breast cancer, to determine whether there is an association between IgG antibodies to CMV and EBV and risk of breast cancer.

## MATERIALS AND METHODS

### Subjects

The Australian Breast Cancer Family Study (ABCFS) is a population-based case–control-family study of breast cancer (Hopper *et al*, 1994; McCredie *et al*, 1998; Hopper *et al*, 1999). For this study, eligible cases comprised women aged under 40 years with a first diagnosis of invasive breast cancer in 1992–1995 reported to the Victorian or New South Wales Cancer Registries. Notification of cancer diagnoses is required by legislation in Victoria and New South Wales. Controls from the electoral rolls were selected by proportional random sampling based on the expected age-distribution of the cases, and were aged under 40 years at invitation into the study. Cases and controls were interviewed in their homes by trained interviewers using the same questionnaire (addressing known and putative risks for breast cancer) for cases and controls. Interviews were conducted for 466 cases (72.5% of those eligible) and 408 controls (64.5% of those eligible). Blood samples were collected from 393 cases and 295 controls, and stored plasma was available for 208 cases and 169 controls (the protocol during the early part of the study did not include storing of plasma). Women for whom stored plasma was available did not differ significantly from those for whom stored plasma was not available with respect to age or any of the measured putative risk factors. Approval for the ABCFS was obtained from the ethics committees of the University of Melbourne and The Cancer Councils of Victoria and New South Wales.

### Measurement of IgG Antibodies

Measurement of IgG antibodies to CMV and EBV was based on 0.4 ml of stored plasma from each woman. Each plasma sample was tested, blind to case–control status, using standard Victorian Infectious Diseases Reference Laboratory (VIDRL) enzyme immunoassays for CMV IgG and EBV viral capsid antigen IgG with measurement in units of optical density (OD). Seropositivity was defined by VIDRL as >0.2 for CMV and ⩾0.2 for EBV.

### Statistical analysis

The difference between means was assessed using the *t*-test and differences in distribution of IgG values by the Wilcoxon rank-sum test. The risk of breast cancer was estimated using multivariate logistic regression with STATA software.

A reference age (age at diagnosis minus 1 year for cases, and age at interview for controls) was used for all analyses (Hopper *et al*, 1999). Adjustment for confounding by reference age, verified history of breast cancer in first-degree relatives, education, country of birth, state, marital status, BMI, height, age at menarche, number of live births, and use of oral contraceptives was made as in previously published case–control analyses (McCredie *et al*, 1998; Hopper *et al*, 1999).

## RESULTS

Table 1

**Table 1:** Seropositivity for CMV and EBV and risk of breast cancer

	**Cases (*N*=208)**	**Controls (*N*=169)**	**Odds ratio^a^ (95% CI)**
CMV positive^b^	122 (58.7%)	97 (57.4%)	1.08 (0.68−1.71)
EBV positive^c^	201 (96.6%)	162 (95.9%)	1.11 (0.34−3.73)

a Adjusted for reference age, education, country of birth, state, marital status, BMI, height, age at menarche, number of live births, ever use of oral contraceptives, and history of breast cancer in first-degree relatives.

b >0.2 optical density units.

c ⩾0.2 optical density units

shows that there was no difference in the prevalence of seropositivity for CMV or EBV between cases and controls (59 and 57% respectively for CMV, *P*=0.8; 97 and 96% respectively for EBV, *P*=0.7). There was no association between risk of breast cancer and seropositivity after adjusting for potential confounders.

Figure 1A

**Figure 1:**
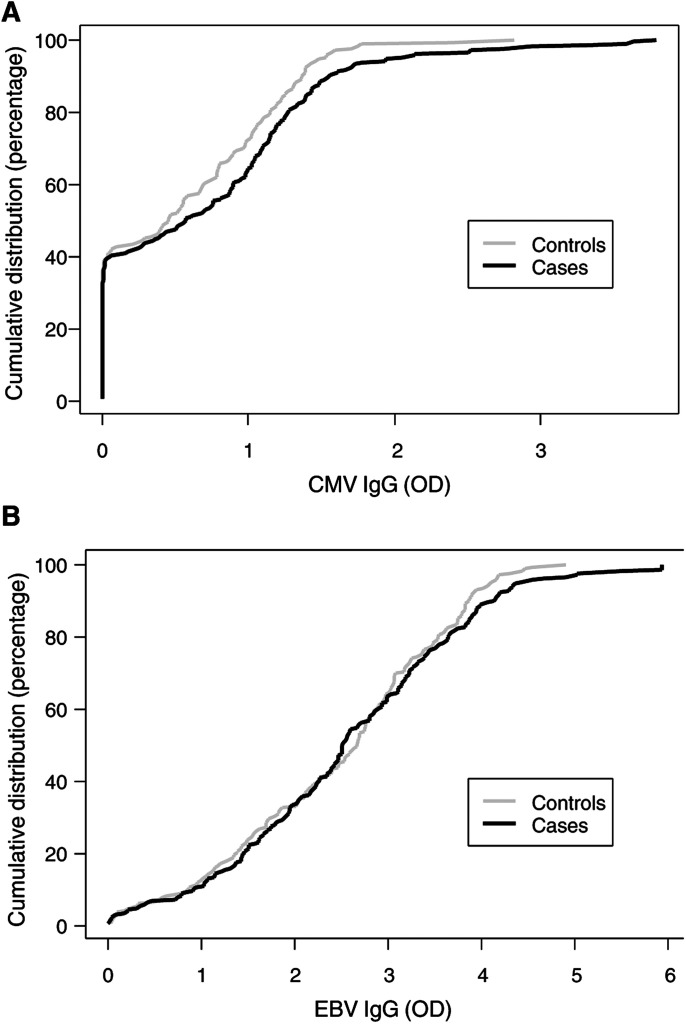
(**A**) Cumulative distribution of CMV IgG values (OD) for controls (grey line) and cases (black line). (**B**) Cumulative distribution of EBV IgG values (OD) for controls (grey line) and cases (black line).

shows that, although the proportion of women who were seronegative for CMV was similar for cases and controls, the cumulative distribution of CMV IgG values for seropositive cases was shifted to the right and had a longer tail than for seropositive controls. Although there was no significant difference in the overall distributions (*P*=0.1), when restricted to seropositive women there was evidence that the distributions for cases and controls differed (*P*=0.02). In seropositive women, mean IgG values were higher in cases than controls for CMV, being 1.20 and 0.98, respectively (*P*=0.005). In contrast, Figure 1B shows that the distribution of EBV IgG values did not differ between cases and controls (*P*=0.7), with the mean EBV IgG values being 2.65 and 2.57, respectively (*P*=0.5).

When the effect of IgG values on risk of breast cancer was examined as a continuous variable, the adjusted odds ratios per OD unit were 1.46 (95% CI 1.06–2.03) for CMV IgG and 1.11 (0.93–1.33) for EBV IgG (Table 2

**Table 2:** CMV and EBV IgG (OD units) and risk of breast cancer

	**Odds ratio^a^ (95% CI)**
CMV IgG	1.46 (1.06–2.03)
EBV IgG	1.11 (0.93–1.33)

a Adjusted for reference age, education, country of birth, state, marital status, BMI, height, age at menarche, number of live births, ever use of oral contraceptives, and history of breast cancer in first-degree relatives.

). Excluding the 18 cases known to carry a germline mutation in BRCA1 or BRCA2 did not change these estimates.

## DISCUSSION

Our study found evidence of an association between CMV IgG levels and breast cancer in young women. The higher CMV IgG levels found in seropositive women with breast cancer could be the result of a more recent infection with CMV (Mendez *et al*, 1999), which would be consistent with the hypothesis that late exposure to CMV is a risk factor for breast cancer. It is also possible, however, that an association between breast cancer and CMV might occur if CMV is a surrogate for late infection by another agent that has similarities to CMV. Our study did not find evidence of an association between EBV IgG levels and breast cancer in young women, although an association between EBV and breast cancer has been the longer established hypothesis (Bonnet *et al*, 1999; Magrath and Bhatia, 1999).

Others have investigated the hypothesis that delayed infection is associated with breast cancer. One study suggested an increased risk of breast cancer associated with late age at onset of self-reported infectious mononucleosis (Yasui *et al*, 2001). The hypothesis has also been investigated by examining birth cohort trends in the incidence of breast cancer and Hodgkin's disease (Krieger *et al*, 2003). Several studies have investigated Epstein–Barr virus gene expression in human breast cancer, with inconsistent results (Bonnet *et al*, 1999; Magrath and Bhatia, 1999; Fina *et al*, 2001; Hermann and Niedobitek, 2003; Xue *et al*, 2003). Our study is the only one we know of to investigate breast cancer and EBV or CMV using blood samples and serological testing.

It is not unreasonable to suggest a viral aetiology for some breast cancers. Several cancers in humans (cervical cancer, liver cancer, and Adult T-cell leukaemia) are known to be caused by viruses, and breast cancer in mice can be caused by a virus; the mouse mammary tumour virus (MMTV) (Hennighausen, 2000). CMV could be associated with breast cancer because it is a ubiquitous virus that is shed in breast milk, as well as in saliva, urine, cervical secretions, and semen, which implies that CMV persistently infects epithelial cells (Sissons *et al*, 2002).

It is possible that chance could explain our findings, even though the difference between cases and controls for mean CMV IgG values and the adjusted odds ratio per OD unit for CMV IgG were both statistically significant. Bias seems an unlikely explanation for our results given the specificity of the association; it is not obvious to us how bias could cause a spurious association between breast cancer and CMV but not between breast cancer and EBV, and antibody testing was carried out by laboratory staff who were blind to disease status. There is potential for confounding by unidentified factors, since this is an observational study, however, adjustment for identified confounding factors made only a small difference to the odds ratios for CMV IgG and EBV IgG, with the specificity of association persisting for CMV IgG and breast cancer.

Not all women with breast cancer in our study had antibodies to CMV (40% of cases in our study had no antibodies to CMV). This is compatible with our understanding that during carcinogenesis a cell must acquire many mutations before it can contribute to tumorigenesis (Knudson, 1971). CMV infection in adult life may play a role in some cells acquiring one or more of the genetic mutations required for the development of breast cancer. An analysis restricted to women without BRCA1 or BRCA2 mutations made no difference to the results; however, only 18 women (all cases) in this study were BRCA1 or BRCA2 carriers.

The main limitation of our study was that it was retrospective, with blood samples collected after the diagnosis of breast cancer. Breast cancer or its treatment may cause disruption to the immune system, leading to increased levels of CMV IgG, but there are no obvious reasons to expect disruption to the immune system that would affect CMV IgG levels but not EBV IgG levels. This specificity of association suggests that the association between CMV and breast cancer could be causal, but further investigations are required, such as prospective studies where blood samples have been taken prior to the diagnosis of breast cancer. Serial samples would provide more information since the interpretation of a single high titre of CMV IgG is difficult; normal immune subjects may have some variation in antibody titre over time, and the effect of reactivation of latent CMV upon IgG titre is unknown (Mendez *et al*, 1999). It would also be important to investigate whether there is any relationship between CMV and breast cancer risk in older women, since our study has only focussed on young women.
